# Neprilysin Inhibits Coagulation through Proteolytic Inactivation of Fibrinogen

**DOI:** 10.1371/journal.pone.0158114

**Published:** 2016-07-20

**Authors:** Matthew Burrell, Simon J. Henderson, Anna Ravnefjord, Fritz Schweikart, Susan B. Fowler, Susanne Witt, Kenny M. Hansson, Carl I. Webster

**Affiliations:** 1 Antibody Discovery and Protein Engineering, MedImmune, Milstein Building, Granta Park, Cambridge, United Kingdom; 2 Biologics Safety Assessment, MedImmune, Aaron Klug Building, Granta Park, Cambridge, United Kingdom; 3 Cardiovascular and Metabolic Disease iMed, AstraZeneca R&D, SE-431 83 Mölndal, Sweden; 4 Pharmaceutical Development, AstraZeneca R&D, SE-431 83 Mölndal, Sweden; National Center for Geriatrics and Gerontology, JAPAN

## Abstract

Neprilysin (NEP) is an endogenous protease that degrades a wide range of peptides including amyloid beta (Aβ), the main pathological component of Alzheimer’s disease (AD). We have engineered NEP as a potential therapeutic for AD but found in pre-clinical safety testing that this variant increased prothrombin time (PT) and activated partial thromboplastin time (APTT). The objective of the current study was to investigate the effect of wild type NEP and the engineered variant on coagulation and define the mechanism by which this effect is mediated. PT and APTT were measured in cynomolgus monkeys and rats dosed with a human serum albumin fusion with an engineered variant of NEP (HSA-NEPv) as well as in control plasma spiked with wild type or variant enzyme. The coagulation factor targeted by NEP was determined using *in vitro* prothrombinase, calibrated automated thrombogram (CAT) and fibrin formation assays as well as N-terminal sequencing of fibrinogen treated with the enzyme. We demonstrate that HSA-NEP wild type and HSA-NEPv unexpectedly impaired coagulation, increasing PT and APTT in plasma samples and abolishing fibrin formation from fibrinogen. This effect was mediated through cleavage of the N-termini of the Aα- and Bβ-chains of fibrinogen thereby significantly impairing initiation of fibrin formation by thrombin. Fibrinogen has therefore been identified for the first time as a substrate for NEP wild type suggesting that the enzyme may have a role in regulating fibrin formation. Reductions in NEP levels observed in AD and cerebral amyloid angiopathy may contribute to neurovascular degeneration observed in these conditions.

## Introduction

Neprilysin (NEP) is an integral type II membrane-bound zinc-dependent peptidase that degrades a number of physiological peptides that are involved in processes such as blood pressure regulation and nociception [1]. NEP has been proposed as a potential therapeutic for the treatment of Alzheimer’s disease because of its activity in cleaving the amyloid-beta (Aβ) peptide [2,3] and studies have shown that increasing the level of brain NEP in animal models significantly reduces brain Aβ [4–7]. We have engineered a variant of NEP (NEP G399V/G714K; “NEPv”) that has increased specificity and activity on Aβ and shown that while peripheral administration of a human serum albumin fusion of this variant (HSA-NEPv) reduces plasma Aβ, the level of the peptide in the brain is not affected [8,9].

While the substrate specificity of the enzyme is broad, NEP has a strong preference for peptides over larger proteins. This preference results from the restricted access of substrates to the enclosed catalytic site [10]. Indeed, until recently it was thought that NEP exclusively cleaved peptides below about 3000 Da. However, recent reports suggest that NEP is also involved in the degradation of protein substrates. For example, NEP is able to cleave fibroblast growth factor-2 (FGF-2), which is a 155 amino acid protein [11]. NEP has also been implicated as a human skin fibroblast elastase [12].

These studies have raised the possibility that NEP may be involved in the degradation of other protein substrates. The most likely candidates for proteolysis by NEP are proteins that contain exposed, flexible regions that are able to access its active site. Fibrinogen, which is converted to insoluble fibrin during coagulation, is a 340 kDa plasma glycoprotein that has a hetero hexameric structure of three polypeptide chains, Aα, Bβ and γ linked by disulphide bonds that contains unstructured flexible regions at the N-termini of its Aα- and Bβ-chains [13]. During the initiation of fibrin formation, the unstructured regions at the N-termini of the fibrinogen Aα- and Bβ-chains, which comprise fibrinopeptides A and B (FpA and FpB), respectively, are released by the serine protease thrombin, exposing the A and B polymerisation knobs that bind to the complementary a and b holes located in the C-termini of the γ and Bβ chains. The knob:hole interactions between fibrinogen monomers allow assembly of the fibrin polymer. Here we report that wild type and an engineered variant of NEP impair fibrin formation and that this effect appears to be mediated by cleavage of the unstructured N-termini of the fibrinogen Aα- and Bβ-chains. Release of FpA by thrombin from a peptide mimicking the NEP-truncated Aα-chain was significantly reduced compared to the full-length substrate, suggesting that NEP exerts an effect on coagulation by causing a reduction in the rate of conversion of fibrinogen to fibrin. Administration of the engineered variant of NEP to cynomolgus monkeys and rats caused increases in *ex vivo* prothrombin time (PT) and activated partial thromboplastin time (APTT) as well as a decrease in plasma fibrinogen concentrations.

## Materials and Methods

### Materials

Human fibrinogen and thrombin were purchased from Sigma-Aldrich (St. Louis, MO). Rat fibrinogen was purchased from Enzyme Research Laboratories (Swansea, UK). Cynomolgus monkey fibrinogen was purchased from Sera Laboratories International (West Sussex, UK). Monkey fibrinogen ELISA kit was from Cusabio Life Science (Hubei Province, China). Thrombin calibrator and platelet poor plasma (PPP) reagent were from Thrombinoscope BV (Maastricht, Netherlands).

### Protein expression and purification

The extracellular domain of human NEP (amino acids 52–749) was expressed as a C-terminal fusion with HSA. G399V/G714K (NEPv) and E584V mutations were introduced into the NEP wild type gene by site-directed mutagenesis. Fusion proteins were expressed in Chinese hamster ovary (CHO) cells as described previously [8]. HSA-NEP proteins were purified from CHO supernatants using affinity chromatography on a column packed with Mimetic Blue (ProMetic BioSciences, Cambridge, UK).

### Animal studies

Animal studies were conducted at Wil Research Europe-Lyon according to protocols reviewed and approved by the ethical committee of the testing facility which is registered with the Ethical Charter of the GRICE group and with CNREEA (National Recognition as Ethical Committee, number 30). The study plans were reviewed by the ethical committee under file number: 58, 2011. The studies were also approved by the MedImmune Institutional Animal care and Use Committee and was in compliance with the AstraZeneca Bioethics policy and the Guide for Care and Use of Laboratory Animals (NRC, 1996). Animals were maintained in an AAALAC-accredited animal facility and non-human primates were housed in groups of 3 in a communal housing compartment (1.80 x 1.20 x 2.10 m) to facilitate socialisation and were only separated for the time necessary to complete study procedures. Monkeys received fruit or vegetables daily in addition to commercial primate diet. Environmental enrichment consisted of music in the animal room and provision of a toy, swings and perches as well as a foraging box containing raisins and sunflower seeds which was provided twice a week. Rats were housed in groups of 5 or 3 and allowed access to pelleted complete diet *ad libitum* except during fasting prior to clinical laboratory determination, urine collection or necropsy. The veterinarian at the testing facility was responsible for providing any medically necessary treatment or euthanasia to prevent unacceptable pain or suffering with palliative or prophylactic care to prevent pain or distress in non-life threatening situations if required. Monkeys were killed at the end of the study by pre-anaesthesia with an intramuscular injection of ketamine hydrochloride (Imalgene 500®, Mérial) followed by an intravenous injection of sodium pentobarbitone (CEVA Santé Animale). Rats were killed by carbon dioxide inhalation and exsanguination. Details of animal studies are given in [9]. Briefly, 1 month repeat dose cynomolgus monkey and rat toxicity studies were conducted by twice weekly intravenous administration (9 doses in total) of HSA-NEPv at 0, 5, 50 and 143 mg/kg in PBS (pH 7.2). Three male animals per dose group were dosed in the monkey study and five male and five female animals per dose group were dosed in the rat study. PT and APTT were measured twice before the start of dosing (Days -10 and -4), on Day 2 (24 hours after the 1^st^ dose) and on Day 32 (3 days after the 9^th^ dose) for cynomolgus monkeys and on Day 27 (1 day after the 8^th^ dose) for rats. Plasma fibrinogen in monkeys was measured using a Fibrinogen ELISA Kit (Cusabio Life Science, Hubei Province, China) according to the manufacturer’s instructions.

### In vitro determination of PT and APTT

For measurement of PT and APTT in plasma samples from rats and monkeys administered with HSA-NEPv, blood was collected into tubes containing trisodium citrate and analysed using an ACL 9000 Autoanalyser (Instrumentation Laboratory, Bedford, MA, USA). PT was measured in recalcified plasma in the presence of exogenous thromboplastin supplied in the PT-Fibrinogen HS Plus kit (Instrumentation Laboratory, Bedford, MA, USA). APTT was measured in recalcified plasma in the presence of synthetic phospholipids and ellagic acid reagent supplied in the SynthASil kit (Instrumentation Laboratory, Bedford, MA, USA).

To determine the effect of spiking exogenous NEP into plasma, PT and APTT were measured for pooled citrated male human, cynomolgus monkey and rat plasma following incubation with 0, 10 or 20 μM NEP protein at 37°C for 6 h using a KC10A Micro coagulometer (Amelung, Lemgo, Germany). APTT was measured by adding 25 μL each of plasma and reagent (PTT Automate, Stago Diagnostica, Asniéres, France) to the coagulometer. After 3 min at 37°C, 25 μL of 25 mM CaCl_2_ was added and the time to coagulation measured. For PT 25 μL plasma was incubated in the coagulometer at 37°C for 1 min after which 50 μL of Thromborel^®^ S was added and the time to coagulation was measured.

### Prothrombinase assay

Prothrombin and prothrombinase activities were determined using the Rox Prothrombin kit (Rossix AB, Mölndal, Sweden) according to the manufacturer’s instructions following incubation of prothrombin with 20 μM NEP protein at 37°C for 6 h.

### Thrombin generation assay

Thrombin generation was measured for pooled human plasma following incubation with 20 μM NEP protein at 37°C for 6 or 16 h using the calibrated automated thrombogram (CAT) assay. Thrombin calibrator or PPP reagent were added to plasma samples in a 96 well plate (Greiner Bio-One) and fluorescence was measured at 37°C with excitation at 390 nm and emission at 460 nm in an Ascent reader (Thermolabsystems OY, Helsinki, Finland). Endogenous thrombin potential was calculated using Thrombinoscope software (Thrombinoscope BV, Maastricht, Netherlands).

### In vitro fibrin formation assays

Fibrin formation assays were performed in 50 mM HEPES (pH 7.4) containing 150 mM NaCl and 1 mg/mL fibrinogen that had been pre-incubated with NEP protein at 0.3–10 μM. Fibrin formation was initiated by the addition of 0.1 U/mL thrombin and 1 mM CaCl_2_ and monitored by observing the change in OD_405_ over 45 min at 37°C on an Envision plate reader (Perkin Elmer, Waltham, MA, USA). To test the effect of NEP on clot lysis by plasmin, fibrin clots were first formed by incubating 1 mg/mL fibrinogen with 0.1 U/mL thrombin and 1 mM CaCl_2_ at in a 100 μL reaction volume 37°C for 25 min. To initiate fibrinolysis, 100 μL 0.1 U/mL human plasmin with or without 6 μM HSA-NEP wild type or HSA-NEPv was added to pre-formed clots and clots lysis was monitored by observing the change in OD_405_ over 60 min at 37°C.

### N-terminal sequencing of fibrinogen chains

Fibrinogen (2 mg/mL) was incubated with 5 μM NEP protein at 37°C and reactions were stopped after 18 h by the addition of LDS Sample Buffer (Life Technologies, Carlsbad, CA, USA). Fibrinogen chains were separated by SDS-PAGE under reducing conditions and transferred to a PVDF membrane. N-terminal analysis was performed on individual bands on an Applied Biosystems 494 HT sequencer (Applied Biosystems, San Francisco, CA, USA) with on-line phenylthiohydantoin analysis using an Applied Biosystems 140A micro HPLC.

### Fibrinopeptide cleavage assays

Fibrinopeptide cleavage by thrombin was measured using a fluorescence polarisation assay [14]. Peptide substrates containing N-terminal 5(6) carboxyfluorescein and C-terminal Biotin (Bachem, Bubendorf, Switzerland) were incubated at 3–400 μM in 50 mM HEPES (pH 7.4) containing 100 mM NaCl and 0.1% bovine serum albumin in the presence of 1–50 U/mL human or rat thrombin at 37°C. Reactions were stopped by the addition of avidin and Complete protease inhibitor (Roche, Basel, Switzerland) and the amount of peptide cleavage was determined by measuring fluorescence polarisation on a Victor plate reader (Perkin Elmer, Waltham, MA, USA).

### Measurement of fibrinogen in plasma samples

Plasma fibrinogen in *ex vivo* samples from cynomolgus monkeys administered with HSA-NEPv was measured using a Fibrinogen ELISA Kit (Cusabio Life Science, Hubei Province, China) according to the manufacturer’s instructions.

### Statistical analysis

All results are expressed as means ± SD, unless otherwise indicated. To compare groups an unpaired, two-tailed, *t*-test with a 95% confidence interval was used (GraphPad Prism, GraphPad Software). The results were considered statistically significant at *P* < 0.05.

## Results

### Effect of NEPv on coagulation in cynomolgus monkeys and rats ex vivo

**Fig 1:**
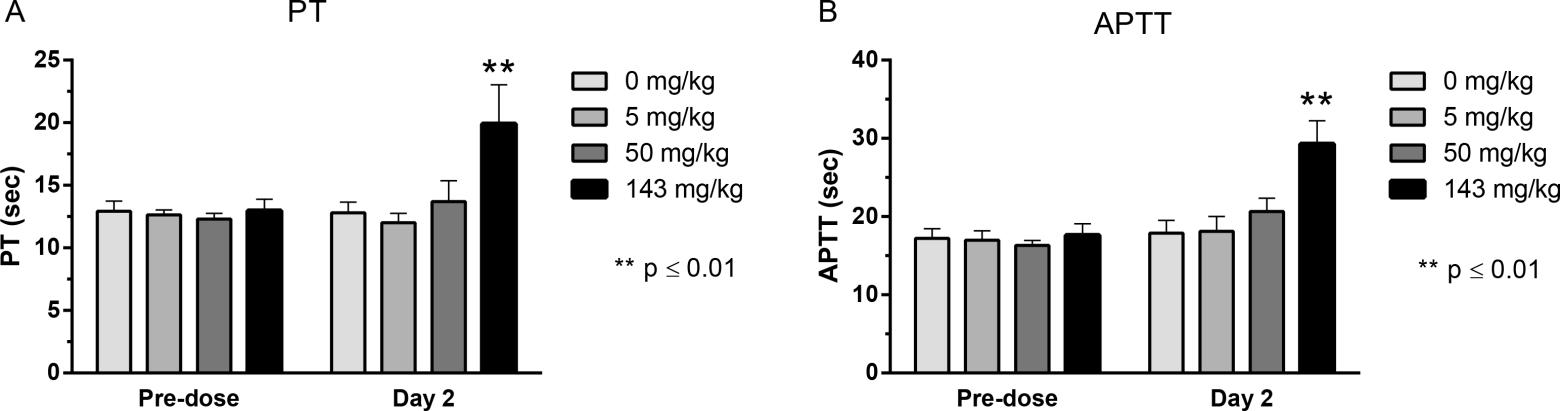
Effect on HSA-NEPv on PT and APTT in cynomolgus monkeys. Mean (± SD) PT (A) and APTT (B) were measured in male cynomolgus monkeys four days before dosing (Predose) and 24 h after administration (Day 2) of HSA-NEPv at dose levels of 0 (vehicle control), 5, 50 or 143 mg/kg (n = 3). **P < 0.01 compared with control.

**Fig 2:**
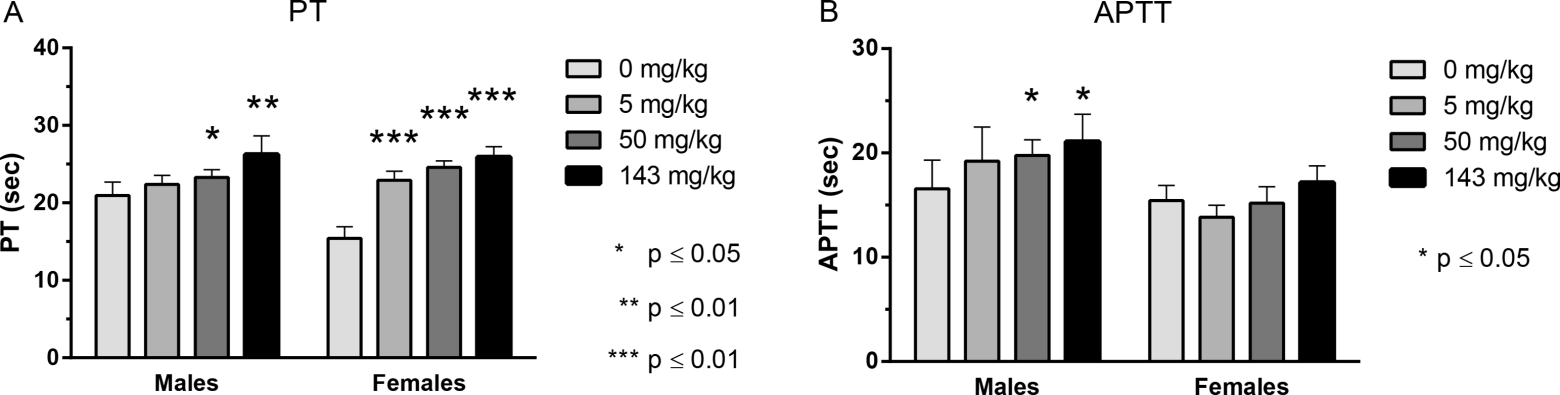
Effect of HSA-NEPv on PT and APTT in rats. Mean (± SD) PT (A) and APTT (B) in rats after twice weekly administration of HSA-NEPv at dose levels of 0 (vehicle control), 5, 50 or 143 mg/kg for 1 month (n = 5 animals/gender/group). PT and APTT were measured on Day 26 (the day after the eighth dose). *P <0.05; **P < 0.01 compared with control.

Previously we have engineered NEP to have increased proteolytic activity on Aβ peptide and tested our improved variant for efficacy in rats and cynomolgus monkeys [8,9]. As part of these studies we investigated the safety profile of HSA-NEPv and observed that the molecule had an unexpected effect on coagulation. Here, we explored this effect on coagulation and found that administration of HSA-NEPv increased both PT and APTT in rats and cynomolgus monkeys compared with controls (Figs 1 and 2). In monkeys, mean PT and APTT increased from 12.8 ± 1 to 19.9 ± 3 s and from 17.9 ± 2 to 29.3 ± 3 s, respectively, the day after the first administration of HSA-NEPv at the highest dose level (143 mg/kg; **P < 0.01). The effect of HSA-NEPv at 50 mg/kg at this time (12.8 ± 1 to 13.7 ± 2 s and 17.9 ± 2 to 20.7 ± 2 s, respectively) was not statistically significant. No effect was observed at the 5 mg/kg dose level. After repeated administration of HSA-NEPv for a month, one of the three animals dosed at 5 and 50 mg/kg and two of the three animals dosed at 143 mg/kg displayed lower than expected plasma drug concentrations on Day 32 (3 days after the last dose), which was consistent with an anti-drug antibody response to the human protein. However, both PT and APTT in the single animal dosed at 143 mg/kg which did not develop an immunogenic response had doubled by Day 32 (data not shown).

In rats, PT and APTT were only measured 1 day after the 8^th^ dose (Day 27) due to blood volume restrictions. At a dose of 50 mg/kg, the mean PT and APTT in males increased from 20.9 ± 2 to 23.3 ± 2 s and from 16.6 ± 3 to 19.8 ± 2, respectively, compared with controls (*P < 0.05). In females at 50 mg/kg HSA-NEPv, PT increased from 15.4 ± 1 to 24.6 ± 1 s compared with controls (***P < 0.001) although there was no apparent effect on APTT. At a dose level of 143 mg/kg, PT increased from 20.9 ± 2 to 26.3 ± 2 s in males (**P < 0.01) and from 15.4 ± 1 to 25.9 ± 1 s in females (***P < 0.001). At this dose level, APTT was not significantly affected in females whereas it increased from 16.6 ± 3 to 21.1 ± 3 s in males (**P < 0.01). The effects on PT and APTT were therefore broadly similar in male and female rats although the effect on APTT was less clear cut in females. The lack of significant effect at lower dose levels is likely to be due reduction in plasma HSA-NEPv levels resulting from an anti-drug antibody response. HSA-NEPv was well tolerated by both monkeys and rats and no adverse clinical signs or macroscopic or microscopic changes were associated with the observed changes in PT and APTT.

### Effect of NEP on coagulation in vitro

To investigate whether NEP wild type has a similar propensity to affect coagulation as NEPv, control cynomolgus monkey, rat or human plasma was spiked with HSA-NEP wild type fusion protein and PT and APTT were determined. Incubations were also performed with HSA-NEPv. An inactive variant of the enzyme, HSA-NEP E584V, was used to exclude the possibility that effects on coagulation were due to an impurity in the protein preparations. NEP proteins were incubated in plasma at 10 and 20 μM so that their concentrations were in a similar range to the peak plasma levels for HSA-NEPv at the 50 and 143 mg/kg doses at which effects on PT and APTT were observed in the *in vivo* studies described above (see S1 Table).

**Fig 3:**
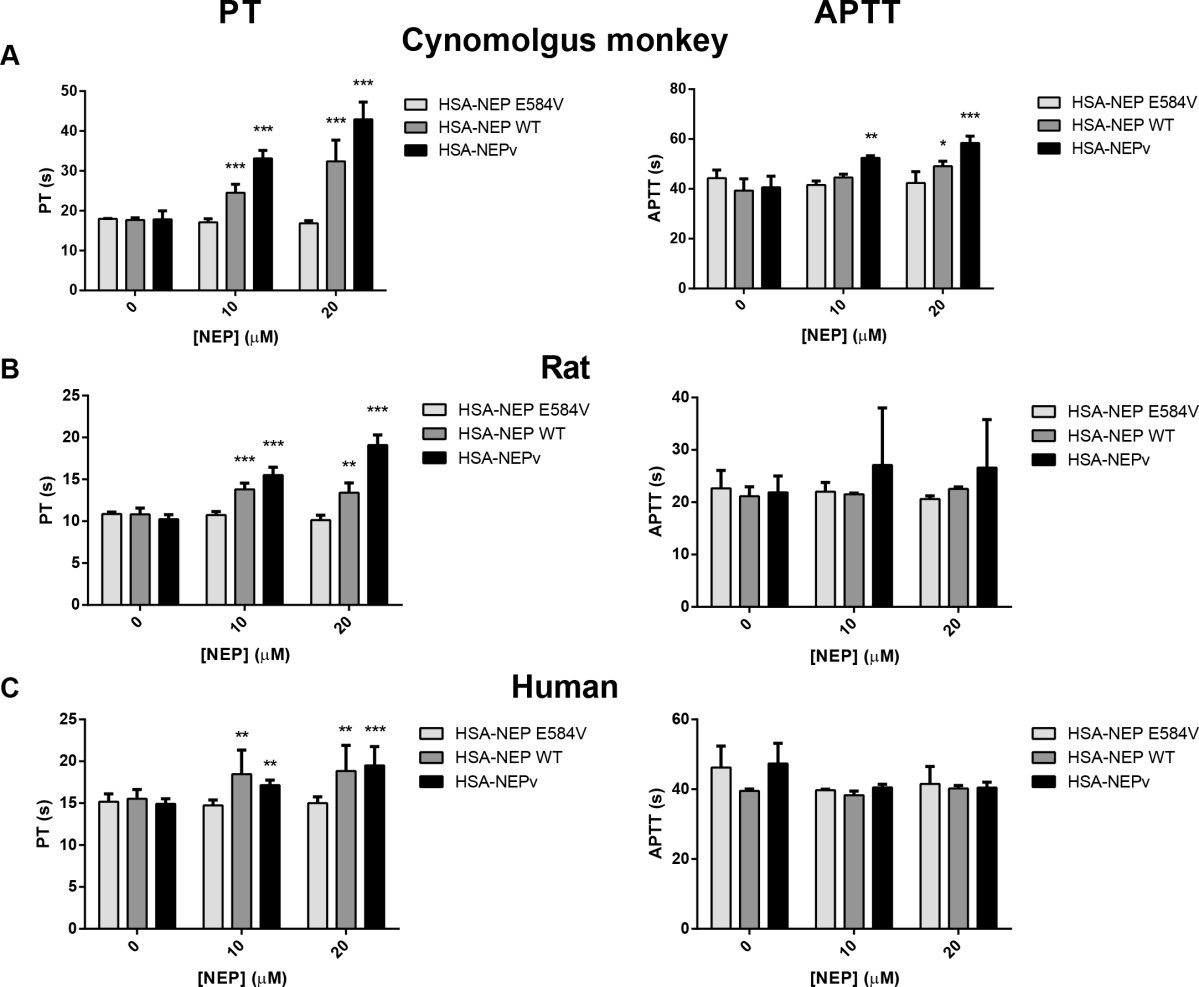
Effect of incubation of NEP in plasma on PT and APTT *in vitro*. HSA-NEP E584V, HSA-NEP wild type or HSA-NEPv were incubated at 0, 10 or 20 μM in cynomolgus monkey (A), rat (B) or human plasma (C) for 16 h after which PT (left panel) and APTT (right panel) were measured (mean ± SD; n = 3). Data shown are representative of two replicate experiments.

Following incubation of cynomolgus monkey plasma with 10 μM HSA-NEP wild type the PT value was increased from 17.8 ± 1 to 24.5 ± 2 s (***P < 0.001; Fig 3A). Raising the concentration of HSA-NEP wild type to 20 μM gave an even greater effect, increasing PT to 32.4 ± 5 s (***P < 0.001). Incubation of cynomolgus monkey plasma with HSA-NEP wild type also caused an increase in APTT from 41.4 ± 4 s to 44.5 ± 1 s (*P* > 0.05) and 49.1 ± 2 s (*P < 0.05) at 10 and 20 μM enzyme, respectively. Even greater effects were observed with HSA-NEPv, which increased PT and APTT from 17.8 ± 1 to 42.9 ± 4 s (***P < 0.001) and 41.4 ± 4 to 58.4 ± 3 s (***P < 0.001), respectively at a concentration of 20 μM.

HSA-NEP wild type also increased PT in rat plasma (Fig 3B). Control rat plasma had a PT value of 10.6 ± 0.6 s; this was increased to 13.4 ± 1 s (**P < 0.01) after incubation with 20 μM HSA-NEP wild type. Again, HSA-NEPv caused a greater increase in PT. Although HSA-NEP wild type and HSA-NEPv appeared to cause slight increases in APTT, the effects observed were not significantly greater than those observed with the inactive variant HSA-NEPv.

To determine whether the effects of NEP on coagulation observed in cynomolgus monkey and rat plasma were likely to translate to man, PT and APTT were measured in human plasma after incubation with the enzyme (Fig 3C). Control human plasma had a PT value of 15.2 ± 0.8 s. This was increased to 18.8 ± 3 s (**P < 0.01) and 19.5 ± 2 s (***P < 0.001) following treatment with 20 μM HSA-NEP wild type or HSA-NEPv, respectively. The effects of HSA-NEP wild type and HSA-NEPv on APTT following incubation for 6 h in human plasma were not statistically significant.

HSA-NEP E584V did not cause any increase in PT or APTT at either 10 or 20 μM in any of the plasma tested, confirming that the effects on coagulation parameters were not due a contamination in the protein preparations.

### Identification of the coagulation factor affected by NEP

The observation that NEP affected both PT and APTT suggested that the molecule was acting on a factor common to both the intrinsic and extrinsic coagulation pathways, which converge on the activation of FX to FXa. Factors common to the two pathways include all components of the tenase and prothrombinase complexes as well as fibrinogen. Since PT is known to be sensitive to FV, FX, FVII, prothrombin and fibrinogen the main focus for investigation were the factors comprising the prothrombinase complex as well as fibrinogen

The possible effect of NEP on prothrombin and prothrombinase complex functional activity was assessed using a prothrombinase assay in which active thrombin formed by the prothrombinase complex is measured by hydrolysis of a chromogenic substrate. Spiking HSA-NEP wild type or HSA-NEPv into this assay at 10 or 20 μM did not reduce the amount of functional thrombin measured relative to control assays using the inactive HSA-NEP E584V, suggesting that NEP does not directly affect prothrombin or any other factor in the prothrombinase complex (S1 Fig).

To further confirm this lack of effect on the prothrombinase complex or any other factor upstream of this complex, endogenous thrombin potential (ETP) was measured on human plasma samples that had been incubated with HSA-NEP wild type, HSA-NEPv or HSA-NEP E584V using the CAT assay. Incubation of plasma with 20 μM HSA-NEP wild type or HSA-NEPv for 6 h caused no statistically significant reduction in ETP compared to control assays with buffer or HSA-NEP E584V. Even with an increased incubation time of 16 h, no significant effects on ETP were observed (S2 Fig).

**Fig 4:**
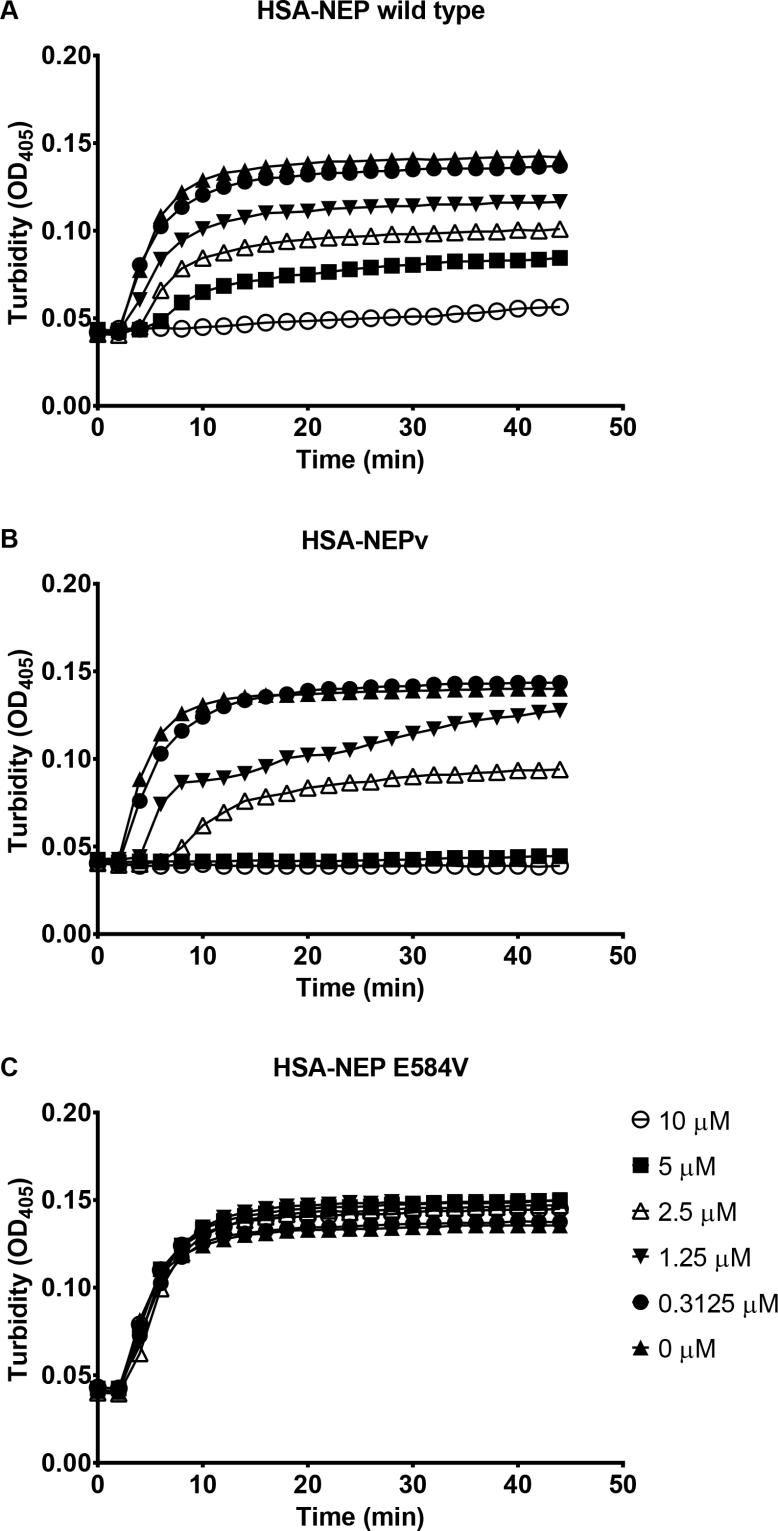
Effect of NEP on fibrin formation. Human fibrinogen was incubated with a range of concentrations of HSA-NEPv (A), HSA-NEP wild type (B) or HSA-NEP E584V (C) for 4 h after which fibrin formation was initiated by the addition of thrombin and CaCl_2_ and OD_405_ was measured for 45 min (mean ± SD; n = 2).

The lack of effect in these assays suggested that impairment of coagulation by NEP was mediated by inactivation of a factor downstream of thrombin. Therefore, we investigated whether NEP could directly affect conversion of fibrinogen to fibrin. When fibrinogen was incubated at 6 μM with HSA-NEP wild type, fibrin formation was inhibited in a concentration dependent manner and was virtually abolished at 10 μM enzyme (Fig 4). HSA-NEPv type also inhibited fibrin formation although the potency was higher than with the wild type enzyme; full inhibition was achieved at 5 μM HSA-NEPv. HSA-NEP E584V caused no effect on fibrin formation, confirming that inhibition caused by HSA-NEP wild type or HSA-NEPv was not due to a contaminant in the protein preparations. To further confirm that this effect was due to the enzymatic activity of NEP, fibrin formation was monitored following incubation of fibrinogen with HSA-NEP wild type or HSA-NEPv in the presence of the specific NEP inhibitors phosphoramidon and thiorphan. Impairment of fibrin formation by HSA-NEP wild type was completely abolished by the addition of these inhibitors (S3 Fig). Phosphoramidon and thiorphan did not completely restore the ability of fibrinogen incubated with HSA-NEPv to form fibrin. This observation, however, is consistent with previous data that showed that these inhibitors are much less potent towards the mutant enzyme compared to NEP wild type.

The results described above suggest that NEP exerts its effect on coagulation by directly inhibiting the conversion of fibrinogen to fibrin. However, it was also possible that the enzyme could have an effect on the fibrin polymer itself. To test this possibility, the ability of NEP to accelerate fibrinolysis by plasmin was assessed. Human fibrinogen was converted to fibrin by thrombin after which plasmin was added to the pre-formed clots with HSA-NEP wild type, HSA-NEPv, BSA or assay buffer. Neither HSA-NEP wild type nor HSA-NEPv caused any acceleration of clot dissolution by plasmin compared to BSA or buffer control assays showing that NEP does not directly affect polymerised fibrin (S4 Fig). In the absence of plasmin no fibrinolysis was observed.

### Mechanism of NEP-mediated inactivation of fibrinogen

The results described above suggested that impairment of coagulation by NEP resulted from proteolytic cleavage of fibrinogen. Given that NEP exhibits a strong preference towards peptides rather than larger proteins, it seemed likely that any cleavage would be directed towards the unstructured N-termini of the fibrinogen Aα- and Bβ-chains FpA and FpB, respectively. To test this hypothesis cynomolgus monkey fibrinogen was incubated with HSA-NEPv or HSA-NEP wild type and N-terminal sequencing was performed on the individual chains.

**Fig 5:**
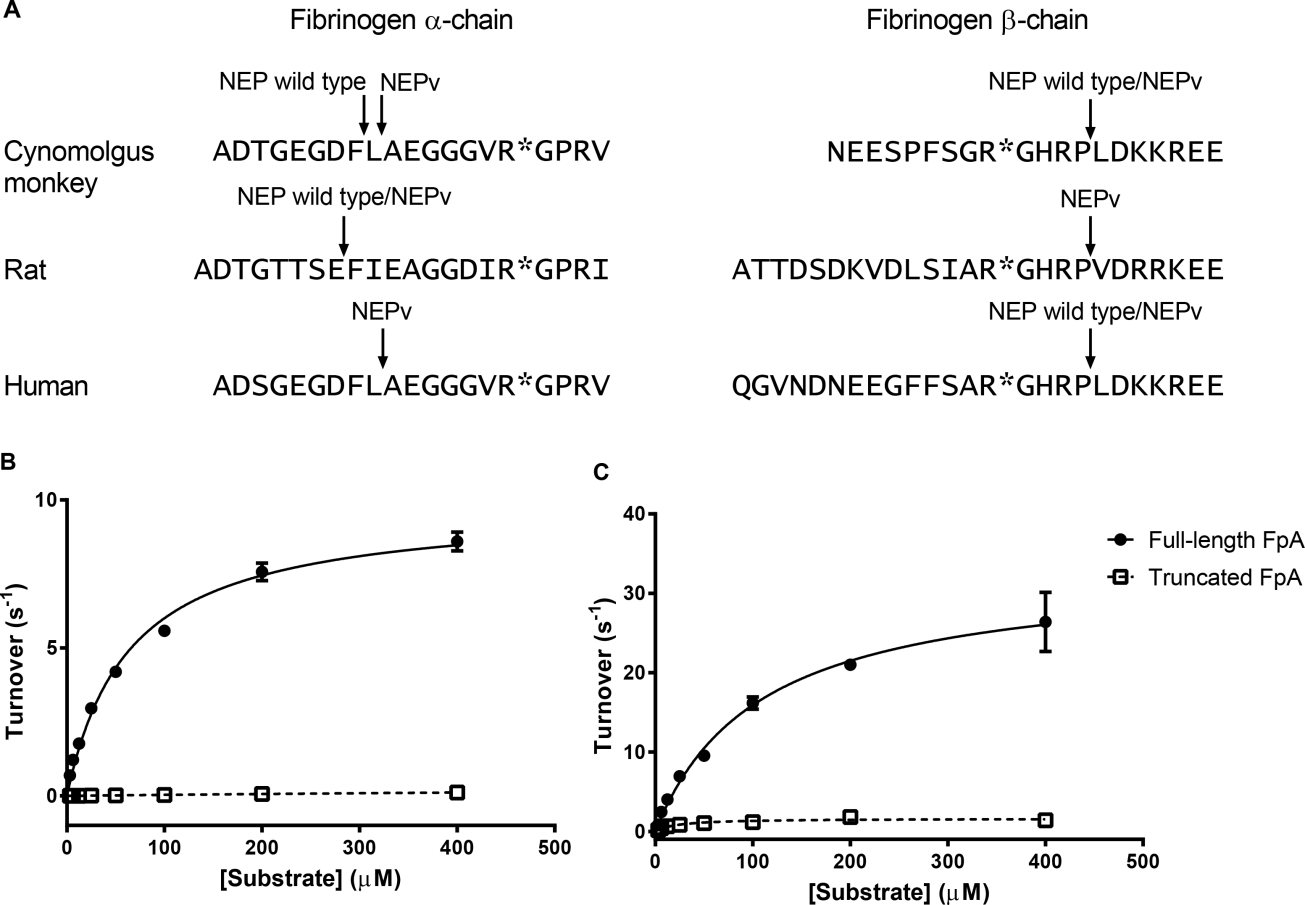
Cleavage sites of NEP wild type and NEPv in fibrinogen α- and β-chains and effect on FpA release by thrombin. Cleavage patterns for NEP wild type and NEPv on human, cynomolgus monkey and rat fibrinogen α- and β-chains were determined by N-terminal sequencing (A). Cleavage sites for NEP are indicated by arrows while those for thrombin are labelled *. Cleavage of full-length and truncated human or rat FpA by thrombin (B and C). Rates of cleavage were determined for full-length (●) or NEP-truncated (□) human (B) or rat (C) FpA by human or rat thrombin, respectively. The Michaelis-Menten equation was used to fit the data. Error bars represent SD (n = 3) and data are representative of two replicate experiments.

N-terminal sequencing of the cynomolgus monkey fibrinogen Aα-chain following incubation with HSA-NEP wild type showed that cleavage occurred at Phe^8^-Leu^9^, removing the first eight amino acids of the chain (Fig 5A). The Aα-chain was also cleaved by HSA-NEPv although in this case cleavage occurred at Leu^9^-Ala^10^. The Bβ-chain was cleaved at Pro^13^-Leu^14^ by both HSA-NEPv and HSA-NEP wild type.

HSA-NEP wild type and HSA-NEPv also cleaved the α-chain of rat fibrinogen. In this case cleavage occurred at Glu^8^-Phe^9^. HSA-NEPv was also able to cleave the rat fibrinogen β-chain although no cleavage of this chain was observed with HSA-NEP wild type.

N-terminal sequencing of the human fibrinogen Aα-chain following incubation for 18 h with HSA-NEP wild type showed that no cleavage of this chain occurred. HSA-NEPv, however, was able to truncate the Aα-chain, cleaving at Leu^9^-Ala^10^. The Bβ-chain was cleaved by both HSA-NEP wild type and HSA-NEPv at Pro^18^-Leu^19^, removing the first 18 amino acids. No cleavage was observed for the fibrinogen γ-chain with either HSA-NEP wild type or HSA-NEPv.

Truncation of the fibrinogen Aα-chain would be predicted to disrupt the interaction of thrombin with FpA, delaying the release of the peptide and the initiation of fibrin formation. To determine the extent of this disruption, cleavage assays were performed on synthetic peptides containing either full-length or NEP-truncated human or rat FpA and the first four residues of the main α-chain (Fig 5B and 5C). k_cat_ for cleavage of the full-length human peptide was 9.1 s^-1^ while K_M_ was 51 μM, giving a k_cat_/K_M_ value of 0.18 μM^-1^s^-1^. These values compared to k_cat_ and K_M_ for release of FpA from native fibrinogen of 59 s^-1^ and 18.7 μM, respectively, reported in the literature [15]. Cleavage of the NEP-truncated FpA synthetic peptide by thrombin was much less efficient compared to the full-length version; k_cat_ was reduced to 0.25 s^-1^ whereas K_M_ increased to 630 μM. Overall, k_cat_/K_M_ was reduced by 490-fold for cleavage of truncated FpA compared to that of the full length peptide. The effect of truncation of rat FpA on cleavage by rat thrombin was much less dramatic; a 5.7-fold reduction in k_cat_/K_M_ was observed for cleavage of truncated rat FpA compared to full-length.

**Fig 6:**
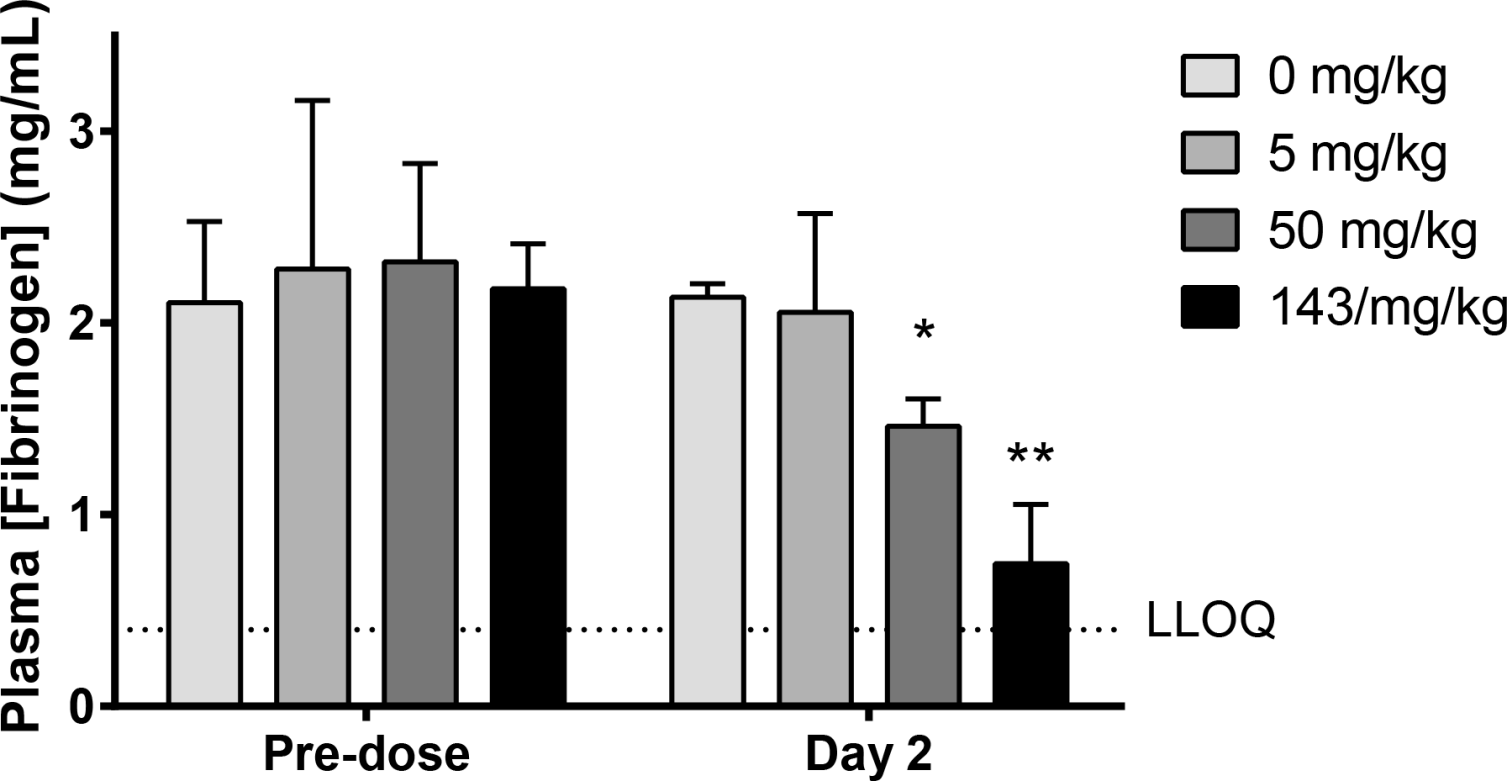
Effect of administration of HSA-NEPv on plasma fibrinogen concentrations in cynomolgus monkey. Fibrinogen concentrations were determined for plasma samples collected four days before dosing (Pre-dose) and 24 h after administration of with HSA-NEPv at 0, 5, 50 or 143 mg/kg (Day 2) (n = 3). LLOQ = lower limit of quantification (0.4 mg/mL). *P <0.05; **P < 0.01.

To determine whether the proteolytic action described above affects fibrinogen levels *in vivo*, concentrations of this coagulation factor in plasma samples from cynomolgus monkeys dosed with HSA-NEPv were measured using an immunoassay (Fig 6). In the animals dosed with 143 mg/kg HSA-NEPv, plasma fibrinogen decreased by 64% from a mean of 2.1 ± 0.07 to 0.75 ± 0.3 mg/mL (**P < 0.01) compared to controls. This decrease in plasma fibrinogen was maintained for the animal that still had drug exposure on day 32 where the plasma fibrinogen concentration was 0.61 mg/mL compared with a mean of 2.1 ± 0.5 mg/mL in controls.

## Discussion

NEP is involved in the regulation of a wide range of physiological peptides, but until recently it was not thought to be active on larger protein substrates. Here, we have shown that NEP wild type causes an increase in PT and APTT in plasma by acting on a factor common to the intrinsic and extrinsic coagulation pathways. Prothrombinase activity and ETP in plasma were insensitive to NEP wild type suggesting that the enzyme exerts its effect on coagulation by directly affecting conversion of fibrinogen to fibrin. This hypothesis was confirmed by the observation that HSA-NEP wild type and the engineered HSA-NEPv impair fibrin formation in assays utilising purified fibrinogen and thrombin. In addition, N-terminal sequence analysis showed for the first time that fibrinogen is a substrate for proteolysis by NEP, adding to a growing number of reports of proteins that are degraded by the enzyme. To date, fibrinogen is the largest molecule that has been identified as a substrate for NEP.

While this study was performed using the recombinant extracellular domain of NEP, and we cannot exclude the possibility that the effect on coagulation is unique to this form of the enzyme, NEP isolated from kidney brush border has comparable kinetic properties to the recombinant protein. For example, NEP from pig kidney and the recombinant human enzyme used in this study display similar kinetic parameters for cleavage of bradykinin, neurotensin and substance P [1,8]. It therefore seems likely the native and recombinant forms of NEP would share similar enzymatic activity towards fibrinogen.

Due to the complexity of the substrate, it was not possible to generate accurate kinetic data for the cleavage of fibrinogen by NEP. However, given that relatively high concentrations of NEP were required to abolish fibrin formation *in vitro*, it appears that catalytic efficiency is significantly lower on fibrinogen than short peptide substrates such as substance P, which is cleaved by recombinant human NEP wild type with a k_cat_/K_M_ of ~ 1 × 10^6^ M^-1^s^-1^ [8]. Nevertheless, NEP is highly abundant in tissues such as the kidney brush border and it is feasible that activity on fibrinogen may be relevant at such sites where high local concentrations of the enzyme exist.

Administration of HSA-NEPv to monkeys and rats confirmed that these effects on fibrinogen can occur *in vivo* and result in increases in PT and APTT as well as reductions in fibrinogen concentration. These studies revealed that the effect of HSA-NEPv on PT and APTT was greater in monkeys compared to rats. It is likely that this difference reflects a greater cleavage activity of the enzyme towards the fibrinogen Aα- and/or Bβ-chains in monkeys compared to rats, resulting from sequence differences between the two species.

Incubation of monkey, rat and human plasma with NEP appeared to have a slightly more pronounced effect on PT compared to APTT. Despite probing the effects of the enzyme on other parts of the coagulation cascade, no factors in addition to fibrinogen were found to be affected by NEP. It is likely that the discrepancy in effects on PT and APTT is due to differences in reagent specificity and is not reflective of a differential effect on the two parameters.

The molecular basis of the inhibition of fibrin formation appears to be cleavage of the N-termini of the Aα- and Bβ-chains of fibrinogen. Cleavage of the Aα-chain by NEP truncates FpA and would be expected to disrupt the interaction with thrombin. Crystallisation of thrombin with a decapeptide analogue of FpA revealed extensive contacts between the peptide and the active site of the enzyme that would be abolished through the activity of NEP [16]. Previous studies have also shown that truncated FpA substrates are cleaved much less efficiently than the full length peptide by thrombin and that the Phe residue in FpA, that occupies the P^9^ position and would be removed by NEP, is critical for efficient cleavage [17,18]. Our kinetic experiments with synthetic FpA that mimics the NEP-cleaved peptide showed that the full-length human FpA is cleaved ~500-fold more efficiently than the truncated version by thrombin.

Cleavage of the fibrinogen Bβ-chain by NEP causes the removal of FpB as well as the GHRP residues that form the B-knob, which interacts with the b-hole. While binding of the B-knob to the b-hole has a lower affinity than the equivalent A:a knob-hole interaction [19], the B:b knob-hole interaction appears to contribute to lateral aggregation and overall stability of the fibrin polymer [20,21]. This interaction is thought to be less critical than the A:a knob-hole interaction in fibrin formation given that mutation of the b-hole has little effect on polymerisation [22]. Cleavage of the Bβ-chain by NEP may therefore affect the stability of fibrin and its subsequent degradation by plasmin *in vivo*.

HSA-NEP wild type showed a strong effect on coagulation in human plasma despite not appearing to cleave the Aα-chain of human fibrinogen. This observation was somewhat surprising given the very high sequence identity of the N-terminus of the human Aα-chain to that of monkey fibrinogen, which was cleaved by the wild type enzyme. Indeed, human and monkey FpA differ only by a single amino acid. It is possible that N-terminal sequencing from a PVDF blot was not a sufficiently sensitive method to detect cleavage in the human fibrinogen Aα-chain by HSA-NEP wild type. The relative contributions of cleavage of the Aα- and Bβ-chains on fibrin formation require further investigation.

It is interesting to note that the engineered variant of NEP appears to have increased inhibitory activity on fibrin formation. As well as its increased activity on Aβ, NEPv also displays a marked change in cleavage site preference. Although wild type NEP acts on a relatively diverse range of peptide substrates it exhibits a strong preference for cleavage on the amino terminal side of bulky hydrophobic residues such as Phe or Leu. We previously showed using mass spectrometry that the engineered enzyme cleaves Aβ1–40 predominantly at Phe^20^-Ala^21^ whereas wild type NEP cleaves the peptide preferentially at Lys^16^-Leu^17^, Leu^17^-Val^18^ and Phe^19^-Phe^20^ [8]. Similarly, the monkey and human fibrinogen Aα-chains were cleaved at the amino terminal of Ala by HSA-NEPv whereas the wild type enzyme cleaved only the monkey Aα chain at the more typical NEP cleavage site of Phe-Leu. It is likely that the altered specificity of NEPv has increased its activity towards fibrinogen and may explain why slightly different cleavage patterns were observed on this protein with wild type and mutant NEP.

The effect of NEP wild type on coagulation in plasma and purified fibrinogen assays implies that the enzyme may have a physiological role in regulating fibrin formation. Evidence from recent studies suggests that this role may be in protecting vessels in the brain from cerebral amyloid angiopathy (CAA). CAA is characterised by the deposition of Aβ in leptomeningeal and cortical blood vessels and its severity and prevalence increases with age and in AD. There is increasing evidence for the role of fibrin(ogen) deposition in the development of CAA from both studies on AD transgenic mice [23–25] and human post mortem brain tissues from AD patients [23,26]. AD patients have significantly more vessels with deposition of fibrinogen co-localised with Aβ [23] and this increased deposition appears to correlate with possession of the APOE e4/e4 allele [26]. Analysis of post-mortem brain tissue showed reduced NEP levels in blood vessels in patients with CAA [27,28], correlating with the severity of the condition [29]. On the basis of these data we suggest that reduced levels of NEP in the brains of such patients may affect deposition of both Aβ and fibrinogen, thereby contributing to CAA pathology.

Aβ42 has been shown to interact directly with fibrin(ogen) near the C-terminus of the Bβ-chain [30] and alter the structure of the fibrin clot *in vitro* making it more resistant to degradation by plasmin [23,31,32]. We suggest that NEP may therefore affect the deposition of fibrinogen in two ways: 1) by reducing the levels of Aβ in vessels and thereby affecting its interaction with fibrinogen and 2) by directly reducing the ability of fibrinogen to form stable clots and so make it less likely that plasmin-resistant altered clot structures are formed.

In conclusion, this work not only identifies fibrinogen as a new substrate for NEP but also raises the possibility of NEP as having a previously unidentified role in the development of CAA. Further investigation is warranted to fully understand physiological relevance of this role. Our findings also have potential implications for the clinical use of NEP inhibitors in cardiovascular disease [33]. The risk of inhibiting the previously unidentified activity of NEP on fibrinogen may require monitoring alongside concerns already raised about such therapies around Alzheimer’s disease and cancer [34].

## Supporting Information

S1 Fig Prothrombinase activity of plasma samples incubated with NEP fusion proteins. Prothrombinase activity in plasma was measured following incubation with 10 or 20 μM HSA-NEP wild type, HSA-NEPv or HSA-NEP E584V at 37°C for 6 h. Mean data are presented with SD (n = 2).
(EPS)

S2 Fig Endogenous thrombin potential (ETP) of human plasma incubated with NEP fusions proteins. ETP was determined for human plasma samples following incubation with 10 or 20 μM HSA-NEP wild type, HSA-NEPv or HSA-NEP E584V at 37°C for 16 h. Mean data are presented with SD (n = 3).
(EPS)

S3 Fig Effect of inhibitors on impairment of fibrin formation by NEP. Human fibrinogen was incubated with 10 μM HSA-NEP wild type or HSA-NEPv in the presence of 0.5 mM phosphoramidon or thiorphan (or 5% DMSO in the control) at 37°C for 4 h after which fibrin formation was initiated by the addition of thrombin.
(EPS)

S4 Fig Effect of NEP on fibrinolysis by plasmin. Fibrin clots were formed in a 96 well plate by adding thrombin to fibrinogen after which HSA-NEP wild type, HSA-NEPv, BSA or buffer were added and fibrinolysis was initiated by the addition of plasmin. Mean data points are shown (n = 2).
(EPS)

S1 Table Mean peak plasma exposure of HSA-NEPv in cynomolgus monkeys and rats. HSA-NEPv plasma concentrations were determined 5 min after the first dose in cynomolgus monkeys (n = 3) and 1 h after the ninth dose in rats (n = 10).
(DOCX)
