# Supplementary material for: Neprilysin Inhibits Coagulation through Proteolytic Inactivation of Fibrinogen
Source: PLoS One. 2016 Jul 20;11(7):e0158114. doi: 10.1371/journal.pone.0158114 (PMC4954676; doi:10.1371/journal.pone.0158114)
Supplement: S1 Table — HSA-NEPv plasma concentrations were determined 5 min after the first dose in cynomolgus monkeys (n = 3) and 1 h after the ninth dose in rats (n = 10). (DOCX) [file pone.0158114.s005.docx]

|  | Peak plasma exposure | | | | | |
| --- | --- | --- | --- | --- | --- | --- |
|  | 5 mg/kg | | 50 mg/kg | | 143 mg/kg | |
|  | µg/mL | µM | µg/mL | µM | µg/mL | µM |
| Cynomolgus monkey | 91.6 | 0.62 | 1250 | 8.5 | 3600 | 25 |
| Rat | 57.2 | 0.39 | 901 | 6.1 | 2990 | 20 |
